# Exercise interventions for mental disorders in young people: a scoping review

**DOI:** 10.1136/bmjsem-2019-000678

**Published:** 2020-05-04

**Authors:** Michaela C Pascoe, Alan P Bailey, Melinda Craike, Tim Carter, Rhiannon Patten, Nigel K Stepto, Alexandra G Parker

**Affiliations:** 1 Institute for Health and Sport, Victoria University, Melbourne, Victoria, Australia; 2 Department of Cancer Experiances, Peter MacCallum Cancer Centre, Melbourne, Victoria, Australia; 3 Orygen, The National Centre of Excellence in Youth Mental Health, University of Melbourne, Parkville, Victoria, Australia; 4 Mitchell Institute, Victoria University, Melbourne, Victoria, Australia; 5 Institute of Mental Health, School of Health Sciences, University of Nottingham, Nottingham, Nottinghamshire, UK

**Keywords:** adolescent, mental, physical activity, well-being

## Abstract

**Aims:**

This scoping review determines the breadth and outcomes of controlled trials testing the effect of physical activity/exercise interventions across mental health outcomes in young people with a mental disorder.

**Methods:**

The literature search was conducted using the open-access ‘Evidence Finder’, a comprehensive youth mental health-specific database that is systematically populated from MEDLINE, Embase, PsycINFO and Cochrane CENTRAL databases.

**Results:**

Sixteen publications were identified after meeting the following eligibility criteria: (1) participants were young people (mean age 12–25.9 years) with a mental disorder diagnosed by a trained clinician or by reaching a predefined cut score on a symptom measure, (2) interventions were exercise, (3) designs were randomised or non-randomised controlled trials, (4) outcomes were mental health related. Eight studies included young people with depression, three included people with psychosis/schizophrenia, three included people with eating disorders and two included people with anxiety. The available evidence suggests that moderate-to-vigorous-intensity exercise may be beneficial, particularly for reducing depression. The available evidence for other intervention intensities, and for other mental disorders, is mixed.

**Conclusions:**

Overall, the evidence regarding the impact of exercise interventions on a range of mental health outcomes in clinical populations of young people with various mental disorders looks promising but requires further development. Findings from this scoping review can inform the development of future exercise interventions in the youth mental health field.

**What is already known?**

- Worldwide, mental illnesses are the leading cause of disability in young people.
- Current evidence-based treatments for mental illness, such as psychotropic medications and specific psychotherapies, are only modestly effective in young people.
- The benefits of exercise on mental health have been well studied in adults, but not in adolescents and young adults.

**What are the new findings?**

- Moderate-to-vigorous-intensity physical activity/exercise may be beneficial for reducing depression in young people.
- The available evidence for other intervention intensities, and for other mental disorders, is mixed.
- The potential impact/benefit of self-selected/preferred interventions, as opposed to prescribed exercise, has not been well studied.

##  Introduction

 More than a quarter of the world’s population is aged between 10 and 24 years^1^,^3^ and it is during this age range that most major mental disorders emerge.^4 5^ Worldwide, mental illnesses are the leading cause of disability in young people,^6^ with at least 25% of young people diagnosed with a mental or substance use disorder annually.^7^ To prevent or minimise functional disability such as poorer social, educational and vocational opportunities and outcomes, there is a need to effectively treat youth mental disorders in a timely manner, which can reduce symptomatology, relapse and the potential persistence of illness.^8^ Additionally, many health-related behaviours, such as physical activity (PA), are established during adolescence and early adulthood,^9^ highlighting that this developmental period is critical for intervention.



Current evidence-based treatments for mental illness, such as psychotropic medications and specific psychotherapies, are only modestly effective in young people (eg, see ref 10,12), more than half of young people with depression fail to respond to the best available guideline-recommended treatment delivered in controlled trials.^10 13^ The former treatments can also have severe side effects, which can be intolerable to young people, and can lead to further health complications such as an increased risk for metabolic syndrome.^14^ For these reasons, youth mental health researchers and practitioners are increasingly interested in alternative treatment methods such as PA and exercise.

### Rationale

PA and exercise are low risk, acceptable and have less stigma attached to them as a treatment modality, in comparison to counselling or psychotherapy, which is important to young people.^15^ PA and exercise also have appeared effective to the general public as an intervention for improving mental health.^16^

The benefits of PA, defined as any bodily movement produced by skeletal muscles that results in energy expenditure, and exercise, defined as the planned, structured and repetitive undertaking of PA for the purposes of maintaining or improving health or skill-related components of physical fitness,^17 18^ have been well studied in adults.^19^,^22^ The benefits of PA and exercise on mental health, however, have not been well studied in adolescents and young adults, who are experiencing a unique developmental and neurodevelopmental period.^23^ The majority of existing studies in youth mental health have focused on depression symptoms, and have shown that PA and exercise appear to improve symptoms (eg, see ref 24 for an overview of the youth mental health evidence base).

We aim to address this gap by providing a comprehensive overview of the evidence for PA and exercise interventions across all mental disorders affecting young people and explore opportunities for translating these interventions into clinical practice.

### Objectives

This review addressed the following research question: What is known from the existing literature about the effectiveness of PA/exercise interventions as a treatment for mental health outcomes in young people? We therefore have selected to conduct a scoping review, the general purpose of which is to identify and map the available evidence. Indeed, scoping reviews are an appropriate tool to determine the scope or coverage of a body of literature on a given topic as well as give clear indication of the volume of literature and studies available. This is appropriate given that evidence regarding the impact of PA/exercise interventions as a treatment for mental health outcomes in young people is emerging and it is still unclear what other, more specific questions can be posed and valuably addressed by a more precise systematic review.^25^

The parameters within the research question were purposefully broad in order to generate wide coverage.^26^ Within this scoping review, the term ‘mental health outcomes’ refers to (1) specific self-reported mental health symptoms collected using specific quantitative outcome measures and/or (2) diagnosis data collected by specific quantitative outcome measures of symptom severity or structured diagnostic interviews.

We aimed of to provide a comprehensive overview of the breadth and outcomes of controlled trial intervention studies testing the effect of PA/exercise across all mental health outcomes in young people diagnosed with a mental health disorder by a trained clinician or reaching a predefined cut score on a symptom measure.

The objectives were:

1. To examine the extent and range of outcomes from PA/exercise interventions for treatment in a youth mental health context.
2. To collate mental health outcome data and present an overview of the impact of PA/exercise across mental disorder and mental health symptomology.
3. To examine mental health outcomes according to the intensity of the PA/exercise programme.

## Methods

### Protocol and registration

The review was conducted and reported in line with the Preferred Reporting Items for Systematic Reviews and Meta-Analyses extension for Scoping Reviews (PRISMA-ScR) guidelines scoping reviews^27^ and follows the five-stage framework outlined by Arksey and O’Malley.^26^ A review protocol for this scoping review was not registered or published as this study was commenced before the publication of the PRISMA-ScR guidelines which recommend registering the review protocol.

### Eligibility criteria

To determine study eligibility, the following criteria were applied to the studies identified in the initial search: patients had a mental disorder diagnosed by a trained clinician or reached a predefined cut score on a symptom measure and mean age 12–25.9 years; the study delivered a PA/exercise intervention as defined above and included a comparison or control condition, which could be a second PA/exercise intervention; the study reported on the quantitative effect of PA/exercise on at least one of the following mental health outcomes:

- Depression diagnosis or symptoms.
- Anxiety disorder diagnosis, symptoms or trait anxiety measures.
- Substance use diagnosis or symptoms.
- Schizophrenia spectrum diagnosis, first-episode psychosis or symptoms.
- Bipolar disorder diagnosis or symptoms.
- Eating disorder diagnosis or symptoms.
- Suicidality and self-harming behaviours.
- Trauma or stressor-related disorder diagnosis or symptoms.
- General psychological distress.
- Well-being/functioning: quality of life (QoL); functioning (social, educational, vocational, employment).

Eligible studies were either randomised controlled trials (RCT) or non-RCTs, published in English. Excluded studies were those that recruited people with specific physical, intellectual or mental disorder that were not the condition being treated with PA/exercise (eg, effects of exercise on depression in people with epilepsy); samples that had not been diagnosed with a mental disorder by a trained clinician or did not reach a defined cut-off score on a scale that indicated a likely disorder, as we have examined the impact of PA/exercise as a treatment for mental disorder in non-clinical samples in our companion paper to this article^28^; dissertations; and studies published before 1980 as youth mental health was not considered a discrete field prior to 1980.^29^

### Information sources

This review sought to explore the effectiveness of PA/exercise interventions on mental health outcomes in a broad sense and in doing so includes a wide set of mental health outcomes based on the major mental health disorders outlined within the Diagnostic and Statistical Manual of Mental Disorders, Fifth Edition.^30^

The search was conducted using the open-access ‘Evidence Finder’, which is a comprehensive database of all available published controlled trials and systematic reviews of interventions in the youth mental health field (https://www.orygen.org.au/Education-Training/Resources-Training/Evidence-Finder). The ‘Evidence Finder’ is an Australian initiative developed by Orygen, The National Centre of Excellence in Youth Mental Health and headspace, National Youth Mental Health Foundation.^24 29^ The searchable database is populated annually using comprehensive and systematic searches of the Embase, MEDLINE, PsycINFO and Cochrane Library databases, coupled with strict and reproducible inclusion criteria to identify studies. For detailed methodology regarding the ‘Evidence Finder’ development and validation see ref 24 29. This recently developed research tool is becoming more commonly used in the research field.^2531^,^33^ The exact search strategy used in the current scoping review is shown in online supplementary table 3. It includes research published from 1980 to 2019 and contains all available prevention, treatment and relapse-prevention studies in young people (mean age 6–25 years), across the following mental illnesses: anxiety, depression, bipolar, eating disorders, psychosis, substance use and suicide self-harm. It contains controlled trials (including RCTs and quasirandomised studies), systematic reviews and meta‐analyses, published in English. Unpublished trials are not included within the Evidence Finder.

### Search

A single author (MCP) conducted a search of the literature using the ‘Evidence Finder’ in July 2018 (updated in May 2019). All studies that had been classified within ‘Evidence Finder’ as ‘Physical activity/Exercise’ and published between 1980 and 2017 were assessed. No other restrictions were applied to the ‘Evidence Finder’ search as the scoping review sought to synthesise a large number of outcomes across multiple mental health illnesses. Moreover, the reference lists of identified literature reviews, systematic reviews and meta-analyses were searched for suitable primary research. In addition, the studies identified using ‘Evidence Finder’, the reference lists of reviews, systematic reviews and meta-analyses retrieved from the ‘Evidence Finder’ were also checked to identify additional relevant studies.

### Selection of sources of evidence

All titles/abstracts were double screened by any two of a group of three independent authors (MCP, AP or MC). All potentially eligible full texts were then independently reviewed by at least two review authors, and potential conflicts were resolved, where necessary, by consultation with a third author (MCP, AP or MC). There were no conflicts.

### Data charting process

Data charting^26^ was undertaken by three review authors (MCP, APB, TC) using a specifically designed data extraction form, which was designed beforehand. The following data were extracted from all included studies: author; year of publication; study design; study location; sample size and patient characteristics; intervention/control condition characteristics; outcome measures and outcome data.

In order to determine the exercise intensity used for each intervention reported, two assessors (NS, RP) independently reviewed each study’s methods and results for objective (heart rate (HR), %maximal HR, %HR reserve, %1-repetition maximum, per cent of maximal oxygen uptake) and subjective (rating of perceived exertion) measures of exercise intensity. Using these measures, the exercise interventions were classified as light, moderate and vigorous-intensity aerobic exercise according to Norton *et al*^34^ or for resistance exercise using Garber *et al*.^35^ In cases where the exercise interventions were poorly described, we attempted, where possible, to estimate an exercise intensity based on the compendium of exercise energy expenditure and therefore interventions were classified as likely light, likely moderate and likely vigorous intensity.^36^

### Data items

The following data were extracted: mental health outcomes assessed, tools used to measure mental health outcomes assessed, country of study origin, setting of study conduct; study design type; participants, sample size, mean age, overall findings, assessment time points, if intention-to-treat analysis was used (as shown in online supplementary table 1), characteristics of the intervention, characteristics of the control group, personnel delivering the intervention, the delivery format and duration and frequency of the delivered intervention (as shown in online supplementary table 2).

### Critical appraisal of individual sources of evidence

As scoping reviews are generally conducted to provide an overview of the existing evidence regardless of methodological quality or risk of bias, the included sources of evidence are typically not critically appraised, as per PRISMA-ScR guidelines, we did however conduct a partial risk of bias assessment based on Cochrane Guidelines.^37^ Using the Cochrane Risk of Bias Tool^38^ on Covidence Online Software (https://www.covidence.org), a single reviewer (MCP) assessed sequence generation, allocation concealment, blinding of assessors, incomplete outcome data and selective outcome reporting. This is shown in online supplementary table 4. In addition, sample size and effect sizes have been reported in online supplementary table 1.

### Synthesis of results

In order to create a meaningful narrative account of the included literature, a ‘descriptive-analytical’ method was applied which involved applying a common analytical framework.^26^ In this instance, the analytical framework was the mental health outcomes. PA/exercise interventions generally vary the dose, where the combination of intensity (light, moderate or vigorous) and duration (min/week) determines the session and intervention dose. Due to heterogeneity of the duration of interventions and that PA/exercise intensity is strongly linked to affective responses and sustainability, we focused on PA/exercise intervention intensity.

## Results

### Selection of sources of evidence

**Figure 1:**
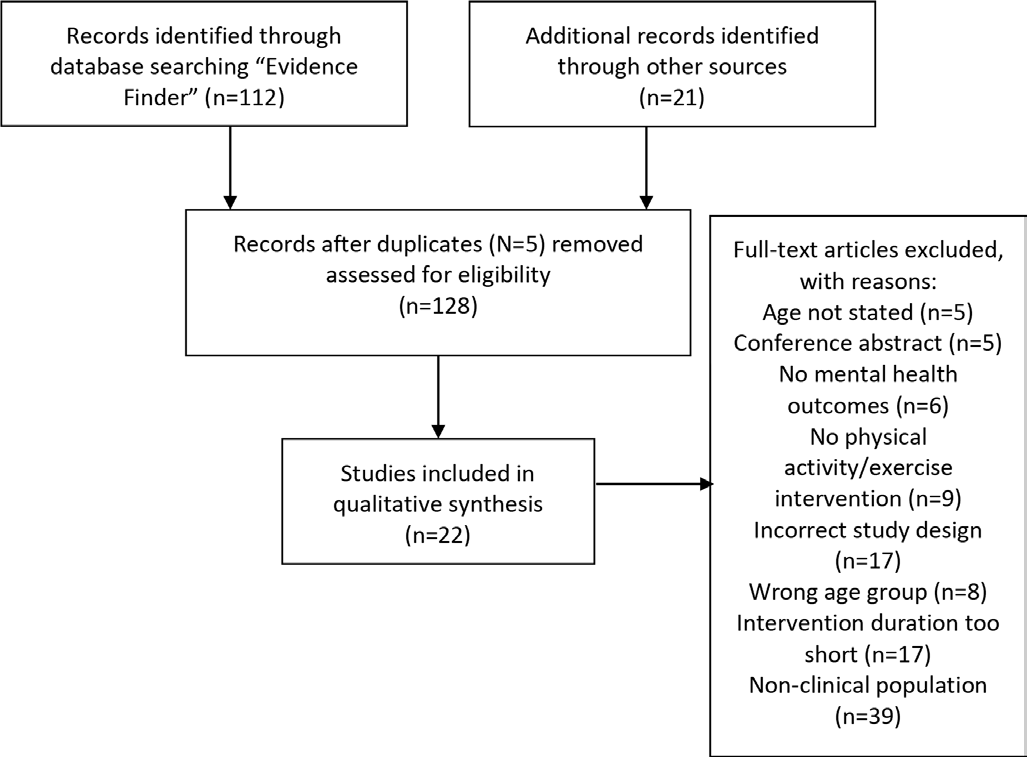
Preferred Reporting Items for Systematic Reviews and Meta-Analyses (PRISMA) flow diagram showing reasons for study exclusions.

A total of 112 records were returned using the ‘Evidence Finder’. Additional articles (n=21) were searched for and identified by going through the retrieved searching reviews, systematic reviews and meta-analyses identified through ‘Evidence Finder’, as shown in figure 1. In total, 22 publications met our inclusion criteria, as shown in online supplementary table 1. Two of these studies^39 40^ report different outcomes from the same trial and therefore we have combined them in our review. Therefore, the current scoping review included 21 trials. While we intended to identify both studies of PA and exercise, all of the included studies are defined as exercise given that the interventions were structured, planned and used to improve health or fitness. Therefore, for the remainder of this paper, we will refer to included studies as exercise-based interventions.

### Characteristics of sources of evidence

**Figure 2:**
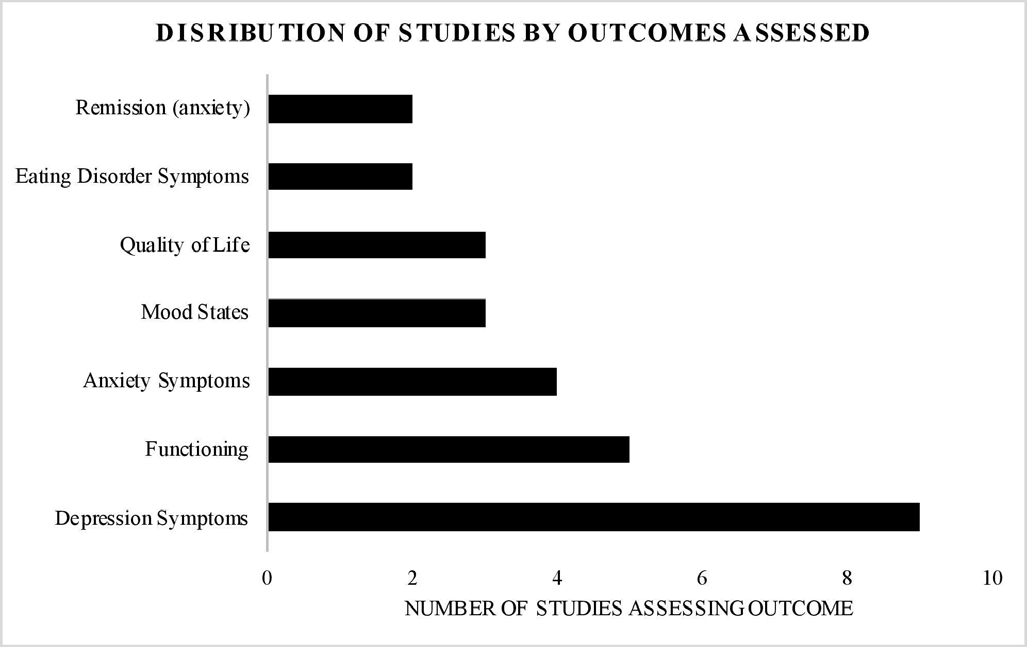
Distribution of mental health outcomes studied. The following outcomes were measured only once and do not appear on the figure: cognition, dysfunctional attitudes, irritability, distress, negative thoughts, psychosis symptoms, remission (depression), self-esteem, substance use, self-efficacy, sleep quality, social adjustment, worry.

**Figure 3:**
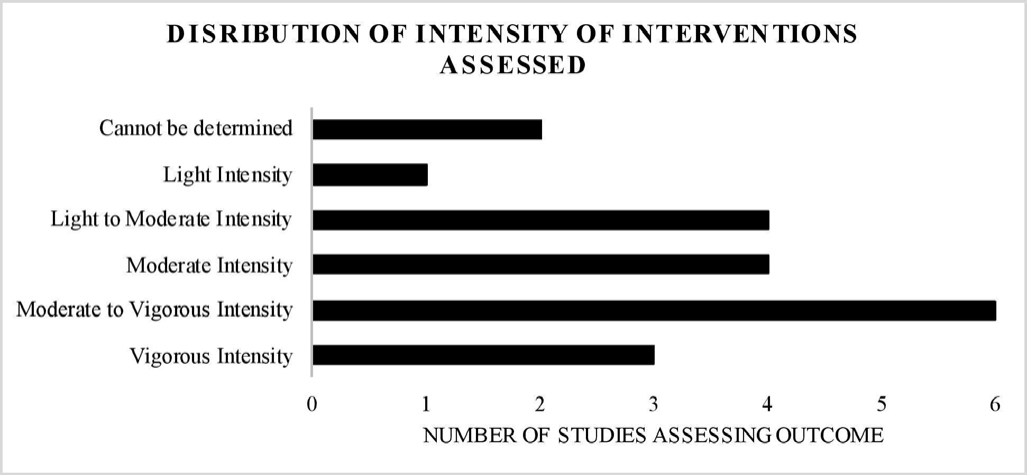
Distribution of interventions studied by intensity.

Only one of the included trials was non-randomised^41^ and one study did not state if it was randomised.^42^ The remaining studies were RCTs. As shown in online supplementary tables 1 and 2, in some studies, participants were randomised to one of two exercise interventions, and therefore there was no non-exercise control group. The findings for each outcome are reported below. All of the included studies assessed interventions that were longer than 3 weeks in duration and assessed sustained mental health outcomes (eg, symptoms of depression), as opposed to state or acute mental health outcomes (eg, state anxiety). Twelve studies included young people with depression, three included people with psychosis/schizophrenia, three included people with eating disorders and two included people with anxiety. Online supplementary table 2 shows that 3 of the studies delivered interventions individually, 6 were in group format, 1 was both in group and individual formats and the remaining 10 studies did not specify the format of intervention delivery. Online supplementary table 2 also shows that eight studies did not specify who delivered the intervention. Figure 2 shows the distribution of mental health outcome studies while figure 3 shows the distribution of interventions studied, by intensity.

### Critical appraisal within sources of evidence

Risk of bias assessments for sequence generation, allocation concealment, blinding of assessors, incomplete outcome data and selective outcome reporting is shown in online supplementary table 4. As shown in online supplementary table 4, high risk of bias was only noted for two of the 22 studies in the domains of incomplete outcome data and selective outcome reporting. Otherwise, all studies scored low or unclear risk of bias in all domains.

### Results of sources of evidence

The relevant extracted data relating to the review questions and objectives are shown in online supplementary tables 1 and 2.

### Types of interventions

Figure 3 shows the distribution of interventions studied by exercise intensity, most commonly delivered in supervised group settings. Moderate-to-vigorous-intensity interventions were the most frequently examined, encompassing a broad range of activities, including strength training, enhanced physical education classes and aerobic exercises. Moderate-intensity interventions formed a considerable portion of those examined, and included predominately aerobic exercises. Light-intensity exercises were also commonly studied, and included yoga, tai chi and stretching exercises. Finally, a smaller proportion of studies examined vigorous-intensity activities, and include predominately aerobic exercises.

### Synthesis of results

#### Depression

##### Depression diagnosis

Depression was the most frequently assessed outcome in this review. Ten studies assessed exercise as a treatment in young people with a clinical diagnosis of depression.^343^,^51^ Four of studies included a non-exercise-based control intervention.^46^,^4850^ Six of these nine studies included individuals with a mean age under 20 years and all but one^50^ found beneficial effects of the exercise as a treatment approach,^4345^,^47 51^ as reported below.

##### Moderate and vigorous-intensity interventions on depression symptoms

One study compared an active vigorous-intensity intervention to a whole body vibration training programme which stimulates a movement pattern similar to human gait but does not require active participation from the individual and found no differences between the groups in depression symptoms or in remission rates.^50^ Another study compared a likely moderate-to-vigorous-intensity intervention to a no-intervention control group, and found that the moderate-to-vigorous-intensity exercise decreased the severity of depression symptoms in male university students with depression.^49^ One study compared two moderate-to-vigorous-intensity interventions, as well as a physical education intervention, the intensity of which was not stated, in female university students with depression. This study found that both of the moderate-to-vigorous-intensity interventions decreased depression from preintervention to postintervention, and that the aerobic exercise decreased depression symptoms compared with the physical education intervention.^51^ Another one study compared a moderate-to-vigorous-intensity intervention to either a cognitive–behavioural therapy (CBT) group, or to a no-intervention control group, and found that both CBT and moderate-to-vigorous-intensity exercise decreased depression in university students with depression, compared with the no-intervention control group; however, the moderate-to-vigorous-intensity intervention did not decrease negative thoughts or dysfunctional attitudes compared with CBT or the no-intervention control group, university students with depression.^48^ Similarly, in another study, a moderate-intensity intervention decreased depression symptoms compared with a no-intervention control group.^43^ Two further studies compared a moderate-intensity with a vigorous-intensity intervention, and found that both intensities reduced depression symptoms from preintervention to postintervention with no differences between the interventions.^44 45^ In one of these studies, both the vigorous and moderate interventions improved social adjustment, psychosocial functioning related to school and relationships with parents and peers, and mood states (anger, fatigue and tension) from preintervention to postintervention^45^; however, neither of these studies included a non-exercise control group.^44 45^

##### Light-to- moderate-intensity interventions on depression symptoms

One study assessed the impact of a preferred intensity intervention (light-to-moderate intensity) and found that it reduced depression symptoms, but only at the 6-month follow-up compared with treatment as usual (TAU), and had no effect on QoL.^46^ In adolescents with depression, a training programme of likely light-to-moderate intensity reduced psychological symptoms and distress compared with no intervention.^52^ A behaviour change intervention encouraging exercise of a self-selected type and intensity also decreased depression symptoms in young people with mild-moderate or subthreshold anxiety and/or depression, compared with psychoeducation, but had no effect on social and occupational functioning, or substance use.^47^ Finally, in young people with mild depression, a likely light-to-moderate yoga intervention decreased depression symptoms, compared with a wait list control group.^3^

##### Summary of findings

Table 1 shows the percentage and frequency of the total number of reviewed studies that showed beneficial effects of a PA/exercise intervention compared with (1) a non-PA/exercise control group intervention, (2) a wait list or no intervention, and (3) compared with a second PA/exercise intervention. Table 2 shows the percentage and frequency of the total number of reviewed studies that showed beneficial effects of a PA/exercise according to the intensity of the delivered intervention.

**Table 1:** Percentage (frequency) of total reviewed studies that showed beneficial effects of exercise interventions compared with a control intervention of depression symptoms

Comparison group	Depression symptoms
Compared with a wait list or no intervention^3 43 49^	100% (n=3 out of 3)^3 43 49^
Compared with a non-PA/exercise control group^46^,^4850^	50% (n=2 out of 4)^4647 77^,^79^
Compared with a second PA/exercise intervention^43 44 49^	(66%) (n=2 out of 3) high and moderate-intensity exercise decreased depression symptoms from preintervention to postintervention, more than a low-intensity exercise intervention.^44^ Moderate-to-vigorous-intensity exercise decreased depression compared with an intervention of unspecified intensity.^80^

PAphysical activity

**Table 2:** Percentage (frequency) of total reviewed studies that showed beneficial effects of exercise interventions depending on the intensity of the intervention

Reported or likely intensity of the intervention	Depression symptoms in people with depression	Depression symptoms in people with a diagnosis other than depression	Anxiety symptoms in people with anxiety	Anxiety symptoms in people with a diagnosis other than anxiety	Eating disorder symptoms	Psychosis/schizophrenia symptoms
Vigorous-intensity intervention	100% (n=2 out of 3)^44 45^	0% (n=0 out of 1)	0% (n=0 out of 1)	None identified	None identified	None identified
Moderate-to-vigorous-intensity intervention	100% (n=3 out of 3)^48 49 80^	100% (n=1 out of 1)^53^	None identified	None identified	33% (n=1 out of 3)^56^	0% (n=0 out of 1)
Moderate-intensity intervention	100% (n=3 out of 3)^43^,^45^	None identified	None identified	None identified	None identified	100% (n=1 out of 1 pre-post analysis only)^58^
Light-to-moderate-intensity intervention	100% (n=2 out of 2)^3 46^	0% (n=0 out of 1)	None identified	0% (n=0 out of 1)	100% (n=1 out of 1)^54^	None Identified
Light-intensity intervention	None identified	None identified	50% (n=1 out of 2)^55^	None identified	None identified	None identified
Variable intensity	None identified	None identified	0% (n=0 out of 1)	None identified	None identified	None identified
Unspecified intensity	None identified	None identified	None identified	None identified	None identified	100% (n=1 out of 1)^42^

##### Other mental health diagnosis

Three studies assessed exercise depression outcomes in young people with a clinical diagnosis other than depression and two of these studies included young people with a mean age under 20 years. In adolescent inpatients with a mean age under 20 years, a running/aerobic exercise programme of likely moderate-to-vigorous intensity reduced symptoms of depression, and improved self-efficacy and mood states (anxiety, hostility, confused thinking and fatigue) compared with standard regularly scheduled exercise classes.^53^ There was no effect in one study of a light-to-moderate-intensity intervention compared with the wait list group in young people with an eating disorder and mean age under 20 years.^54^ A vigorous-intensity intervention did not reduce depression symptoms or worry in young women with generalised anxiety disorder, compared with a wait list control group, but did improve mood states (anxiety-tension) compared with a light-intensity intervention.^40^ None of the studies included an active non-exercise-based control group.

### Anxiety

#### Anxiety diagnosis

Three studies assessed the effects of exercise in young people with anxiety disorders; two of which included an active non-exercise-based control group.^47 55^ One of the studies that included an active non-exercise-based control group assessed young people with a mean age under 20 years, and found that a behaviour change intervention encouraging variable intensity exercise did not reduce anxiety symptoms compared with psychoeducation in people with mild-moderate or subthreshold anxiety and/or depression.^47^ The second study included individuals with a mean age above 20 years, and found that a light-intensity intervention did reduce anxiety symptoms in those with a clinical diagnosis of anxiety, as defined by the Chinese Classification and Diagnostic Criteria of Mental Disorder, compared with counselling.^55^ This study focused on engaging participants in collective outdoor games, which aimed to increase team cooperation.^55^ In contrast, neither a light-intensity nor vigorous-intensity intervention delivered indoors reduced anxiety compared with a wait list control group,^40^ Finally, in regard to remission, a vigorous-intensity intervention, but not a light-intensity intervention, reduced remission rates and irritability in women with generalised anxiety disorder, compared with a wait list control group.^39^

#### Other diagnosis

One study assessed the impact of exercise on anxiety symptoms in young people with a mean age under 20 years and a clinical diagnosis other than anxiety. Anxiety symptoms did not decrease following a likely light-to-moderate-intensity intervention that was delivered individually to young people with eating disorders compared with a wait list control group.^54^ One study assessed the impact of exercise on anxiety symptoms in young people with a mean age over 20 years and a clinical diagnosis other than anxiety. In this study, anxiety symptoms decreased following a likely light-to-moderate-intensity intervention that was delivered in a group setting to young people with depression, compared with a wait list control group.^3^

### Eating disorders

Two studies assessed eating disorder symptoms and a third study assessed QoL in young people with an eating disorder. In one of these, a likely light-to-moderate-intensity intervention decreased eating disorder symptoms and food preoccupation among adolescents (mean age under 20) diagnosed with eating disorders (anorexia nervosa, bulimia nervosa, eating disorder not otherwise specified), compared with a wait list control group.^54^ In the second study, which included young people with a mean age above 20 years, the moderate-to-vigorous-intensity intervention reduced laxative use and, at follow-up, reduced drive for thinness and bulimic symptoms in adolescents with bulimia nervosa, compared with CBT.^56^ A third study found no effect of a moderate-to-vigorous-intensity intervention on QoL in young people with anorexia, compared with no intervention.^57^

### Psychosis/schizophrenia

One study found that a moderate-intensity intervention improved psychiatric symptoms, personal and social functioning and QoL in incarcerated young people with a diagnosis of schizophrenia, from preintervention to postintervention.^58^ In individuals with first-episode psychosis, an intervention of unspecified intensity improved cognitive functioning, school/work functioning, independent living skills and functioning in family relationships, compared with TAU; however, it is unclear if this study included randomised allocation to interventions.^42^ A moderate-to-vigorous-intensity intervention did not improve sleep quality, self-esteem or social, occupational and psychological functioning in people with first-episode psychosis compared with TAU.^41^

## Discussion

This is the first review to determine the breadth and outcomes of controlled trials testing the effect of exercise interventions across mental health outcomes in young people diagnosed with a mental disorder. We have presented an overview of the impact of exercise interventions across mental health symptomology and examined mental health outcomes according to the intensity of the exercise intervention. Depression was the most commonly studied outcome, followed by anxiety and mood states. The most common exercise interventions studied were of a moderate-to-vigorous intensity, followed by light-to-moderate intensity.

### Summary of evidence

#### Moderate-to-vigorous-intensity interventions

The limited available evidence suggests that moderate-to-vigorous-intensity interventions may be beneficial for young people with a mental disorder, particularly for reducing depression symptoms.^43^,^4549 51 53^ These findings are consistent with research in adult populations showing that moderate-to-vigorous exercise is good for mental health.^59^ Some evidence indicates that an effective moderate-to-vigorous-intensity intervention for depression also improved mood states,^45 53^ which may be an important clinical consideration in that immediate changes in mood states may increase motivation or adherence to continue engaging in exercise.^60^ This is particularly relevant as more than half of young people with depression fail to respond to evidence-based, guideline-recommended treatments, such as antidepressants and psychotherapies.^10 13^ Limited evidence also shows that a moderate-to-vigorous-intensity intervention reduces symptoms in adolescents with bulimia nervosa, compared with CBT,^56^ but has no effect on QoL in young people with anorexia, compared with no intervention.^57^ It is possible that exercise and PA are beneficial for bulimia but less so for anorexia nervosa as excessive exercise is a symptom of anorexia nervosa but not necessarily for bulimia.^61^ It is possible that exercise and PA interventions may not be appropriate for mental illness that are characterised by excessive exercise. Overall, however, the results of the current scoping review indicate that PA interventions, where relevant and appropriate, may be a valuable adjunct or treatment option for youth mental health issues.

Only a single study examined a vigorous-intensity intervention on anxiety symptoms, and while there was no significant reducing in anxiety symptoms following resistance exercise or cycling group compared with the wait list control group, the effect size for a reduction in anxiety symptoms following weight training was 0.52 and was 0.54 following cycling. Given that there were only 10 participants in each group, and the effect size was moderate, it is possible that these vigorous-intensity exercises might be found to significantly decrease anxiety symptoms in a larger study.^39 40^

#### Light-to-moderate-intensity interventions

The evidence regarding whether light-to-moderate-intensity interventions reduce depression symptoms is inconclusive, with one study showing positive effects^46^ and three studies indicating no effect.^40 44 54^ An important distinguishing factor of the effective light-to-moderate-intensity intervention is that it was an individualised exercise programme,^46^ where patients could select the intensity or type of exercise in which they engaged. The current scoping review identified two other individualised interventions measuring depression symptoms, both were of unspecified/variable intensity, and both decreased depression symptoms^41 47^; however, it should be noted that one of these studies was not randomised.^41^ This suggests that delivery of tailored or individualised interventions that are based on young people’s preferences may be an important component of exercise interventions that is linked to beneficial outcomes.

There are several explanations for why individualised interventions might be associated with reduction in depressive symptoms. Self-selection of exercise and level of intensity may lead to greater mastery of the activity and thus increase self-efficacy. We found one study that showed improvements in depression corresponded with improvements in self-efficacy, indicating that self-efficacy may partly explain the effect of exercise on depression,^53^ which is consistent with previous research showing that a bidirectional relationship exists between low levels of self-efficacy and elevated depressive symptoms in young people^62^ and highlights that clinical exercise interventions should be designed to be achievable and aim to improve patients’ sense of self-efficacy. Self-efficacy is also central to adherence to exercise. Studies in non-clinical samples of young people show self-efficacy partly mediates the effect of exercise interventions on behaviour.^63^ The importance of self-selection of activity and intensity is also supported by self-determination theory, in which autonomy is proposed as one of three basic psychological needs fundamental to positive mental health.^64 65^ Finally, when intensity is self-selected, rather than imposed, participants experience a greater tolerance to higher intensity exercise.^66^ Therefore, participant-driven preference is an important factor to consider in terms of both mental health outcomes and adherence to exercise, when designing clinical exercise interventions.

The evidence regarding the effectiveness of light-to-moderate-intensity interventions to reduce anxiety symptoms is inconclusive with one study showing positive effects^55^ and two studies indicating no effect.^40 54^ This is consistent with the findings of a previous review in adult populations with anxiety, showing that there is currently insufficient evidence to recommend the frequency, type and intensity of exercise interventions for the treatment of anxiety and therefore the authors recommended exercise engagement according to current health guidelines.^67^ In the current scoping review, the single effective intervention for anxiety focused on engaging participants in collective outdoor games, which aimed to increase team cooperation,^55^ while the non-effective interventions were delivered indoors.^40 54^ This is consistent with previous research showing that undertaking the recommended weekly amount of exercise in outdoor settings predicted lower somatic anxiety, whereas indoor exercise predicted higher somatic anxiety.^68^ Furthermore, one of the non-effective interventions was delivered individually, rather than in a group format.^54^ Previous research indicates that team games/sports may improve psychosocial health in young people above and beyond improvements attributable to participation in exercise, due to the social nature of participation.^69^ Therefore, when designing and implementing exercise-based interventions for young people with anxiety symptoms, it may be beneficial to consider activities that are group based and delivered outdoors; however, this requires further empirical examination to confirm these benefits.

### Strengths and limitations

The strengths of the present study are: (1) this is the first review of such a broad range of mental health outcomes for clinical populations of young people; (2) the inclusion of intervention effects and a synthesis of intervention characteristics potentially driving effects (ie, intensity); (3) the review was conducted in concordance with an established scoping review framework^26^ and reported as per PRISMA-ScR^27^; and (4) the identification of the distribution of the evidence base for exercise interventions across all mental disorders affecting young people. This has shown where we have evidence for exercise interventions for specific mental disorders, and where more research is needed.

There are a number of limitations to consider. Of the 16 studies included in the current review, only six included a follow-up assessment,^4546 53^,^56^ and therefore, the potential long-term benefits of exercise are largely unknown. Only eight^4142 46^,^48 55 56 58^ studies included a non-exercise-based comparison group, while eight studies included a wait list or an additional exercise comparison group. Therefore, in half of the studies reviewed, it is unknown if the observed effects result from the exercise intervention, or from non-specific factors other than the intervention, such as time/attention effects.^70^ Eight studies did not provide sufficient information to determine the intensity of the delivered interventions^4248 52^,^55 57 58^ and eight did not specify the format of intervention delivery.^39 40 42 44 45 48 52 53^ This indicates a need for better specification of intervention intensity and delivery format ensuring clear statements that detail the frequency, intensity, time and type principles of exercise recommendations and prescriptions. We also suggest that reporting should follow the template for intervention description and replication (TIDIER) template for intervention description and replication.^71^ While we intended to identify both studies of PA and exercise, all of the identified studies delivered exercise as an intervention, and therefore the effects of PA of mental health in young people remain unknown. In the current study, the exercise interventions are described only in terms of intensity; however, there may be other potentially important aspects to dose such as total duration, which we have not considered. It would be valuable to discuss how the duration of the various interventions might impact on findings; however, given that the studies identified in the current scoping review vary in terms of intensity of exercise, type of exercise, population studied and outcomes assessed, we believe that there is currently too much variability in the included studies to make any meaningful assessments regarding the impact of intervention intensity on the short-medium or longer term benefits. Furthermore, we only included studies published in English, and therefore might have missed research published in other languages, which could potentially limit the applicability of the findings of the current scoping review. Our included age range of 12–25.9 years is quite large, and this might limit the applicability of the findings of this scoping review. It is possible that teens and young adults respond differently to PA and exercise, and therefore it would be valuable to further explore possible age-related differences among young people in future research. Finally, at the time of the last search, the ‘Evidence Finder’ database included research published between 1980 and 2018. It is possible therefore that some recent publications were relevant to the scoping review, and published in 2019, and not yet have been included in ‘Evidence Finder’ and therefore were missed in our search strategy.

### Future research and practice

This scoping review demonstrates that the evidence currently available is too sparse and heterogeneous to justify applying meta-analytic methods to investigate the effect of exercise on mental health outcomes in young people diagnosed with a mental disorder, with the exception of depression where meta-analysis has previously been conducted.^72^ There are, however, some existing RCT data that in combination with future research, have the potential to allow for more definitive conclusions regarding the effect of exercise on mental health outcomes in young people diagnosed with a mental disorder. Furthermore, the ‘Evidence Finder’ does not include studies including individuals with personality disorders, so we do not know if any primary research exists regarding the impact of exercise in people with personality disorders, and therefore further work should be conducted to present an overview of the impact of exercise in people with personality disorders. Finally, there are challenges to recruiting and engaging young people with a mental disorder into exercise interventions, as factors such as low mood, stress and lack of support are prevalent barriers to engagement.^73^ In order to facilitate uptake and participation, a recent Lancet Psychiatry Commission recommended that lifestyle interventions in mental health settings should partner with trained physical health professionals, and apply principles of behaviour change where required.^74^ We suggest that multidisciplinary approaches be adopted, whereby all treating clinicians are involved in behaviour change interventions to promote uptake of PA or direct delivery of exercise programmes, in order to address some of the challenges to recruiting and engaging young people with a mental disorder into exercise interventions. Encouraging exercise is mentioned in the UK National Institute for Health and Care Excellence guidelines for managing depression in young people but this is only as a consideration rather than a guideline.^75^ In Australia, there are no current guidelines for youth depression. In the Orygen Australian guidelines on prevention and treating psychosis in young people,^76^ exercise is discussed in the context of preventing weight gain from antipsychotic medication; however, PA/exercise are not currently incorporated into the practice management guidelines for youth mental health disorders.

## Conclusions

This review assessed the breadth and outcomes of controlled trials that examined the effects of exercise interventions across all mental health outcomes in clinical populations of young people. We found some evidence that light-to-moderate exercise intensity interventions decreased anxiety symptoms, particularly those delivered outdoors, and in group formats. There was some evidence that light-intensity and moderate-to-vigorous-intensity interventions decreased depression, potentially through improvements in self-efficacy. Few studies have included patient preference for exercise type and intensity when designing clinical interventions. This could be important considering such an approach may facilitate engagement, improve adherence and potentially effectiveness compared with exercise approaches using prescribed type and intensity. The effects of exercise interventions on other mental health outcomes outside of anxiety and depression have only been assessed in single studies to date. This suggests that studies are needed investigating the effects of exercise for young people with psychosis, substance use, bipolar and eating disorders, as well as more across the range of anxiety disorders. We also recommend that future studies include longer term follow-up assessments and an active control group not engaging in exercise. Furthermore, studies should clearly specify descriptions of the exercise interventions detailing the type, intensity, frequency and duration for replication purposes, supported with appropriate quantifiable measures (eg, HR, rate of perceived exertion). The findings from this scoping review will be valuable for the design of future exercise intervention studies, building on the evidence in depression and expanding to include quality research in all mental disorders affecting young people.

## supplementary material

10.1136/bmjsem-2019-000678 online supplementary file 1

## Data Availability

Data are available upon request.
