# Supplementary material for: Exercise interventions for mental disorders in young people: a scoping review
Source: BMJ Open Sport Exerc Med. 2020 May 4;6(1):e000678. doi: 10.1136/bmjsem-2019-000678 (PMC7228557; doi:10.1136/bmjsem-2019-000678)
Supplement: online supplementary file 1 [file bmjsem-6-1-s001.docx]

Table 1. Characteristics of included studies

| **Outcome** | **Study** | **Country** | **Setting** | **Study design** | **Participants** | **Findings [effect size]** | **PA/E group** | **Non PA/E group** | **Assessment Time Point** | **ITT** |
| --- | --- | --- | --- | --- | --- | --- | --- | --- | --- | --- |
| Anxiety Symptoms | Carei 2010(1) | USA | Community | RCT 2 groups | Adolescents diagnosed with eating disorders (n=53) (M age=16.5yrs) | Yoga and WL decreased anxiety (PP) [trait 0.38b; state 0.2b] | Yoga (n=26) | WL (n=27) | PP, FU (4wks) | No* |
|  | Herring 2011, 2011a(2, 3) | USA | Community | RCT 3 groups | Women with a DSM-IV diagnosis of GAD (n=30) (M age=23.5yrs) | No effect [RE-weight training 0.52; AE-cycling 0.54] a | RE-weight training (n=10); AE-cycling (n=10) | WL (n=10) | PP | NS |
|  | Parker 2016(4) | Australia | Community | 2x2 Factorial RCT 4 groups | Young people with mild-moderate anxiety and/or depression (n=176) (M age=17.6yrs) | No effect [nr] | PA + PST (n=44); PA + SC (n=45) | PsyEd + PST (n=43); PsyEd + SC (n=44) | PP | Yes |
|  | Woolery, 2004(5) | USA | Community | RCT 2 groups | Volunteers with mild levels of depression BDI score between 10-15 (n=28) M age=21.5) | Yoga reduced anxiety (BG) [NR] | Yoga (n=13) | WC (n=15) | PP | NS |
|  | Yang 2015(6) | China | Community | RCT 2 groups | University students diagnosed with anxiety disorder (n=38) (M age=21.8yrs) | PA-CRT+ Counselling reduced anxiety (BG) [nr] | PA-CRT+Counselling (n=19) | Counselling (n=19) | PP, FU (3mths) | Yes |
| Cognition | Ventura 2013(7) | USA | Community | CT 2 groups (randomisation NS) | Individuals with first-episode schizophrenia (n=15) (M age=21yrs) | CTr+E improved cognitive functioning (BG) [0.43] | CTr+E (n=ns) | TAU (n=ns) | PP | NS |
| Depression Symptoms | Balchin 2016(8) | South Africa | University | RCT 3 groups | Moderately depressed male university students & staff (n=30) (M age=25.4yrs) | AE-high & AE-mod decreased depression (PP; not BG) [nr] | AE-high (n=9); AE-mod (n=11); AE-low (n=10) | None | PP | Thesis reports 33 randomised, paper reports 30 randomised |
|  | Brown 1992(9) | USA | Psychiatric facility | RCT 2 groups | Adolescents with dysthymia & conduct disorder (n=27) (M age=15.6yrs) | AE-Running decreased depression for girls at 4 weeks (BG) [nr] | AE-running (n=17); Standard PE (n=10) | None | PP, FU (4wks) | No |
|  | Carei 2010(1) | USA | Community | RCT 2 groups | Adolescents diagnosed with eating disorders (n=53) (M age=16.5yrs) | Yoga and WL decreased depression (PP) [0.26b] | Yoga (n=26) | WL (n=27) | PP, FU (4wks) | No* |
|  | Carter 2015(10) | UK | Community | Pragmatic RCT 2 groups | Adolescents scoring >14 on the CDI-2 (n=87) (M age=15.4yrs) | CE-Circuit-training decreased depression at 6mo FU (BG) [nr] | TAU + CE-circuit training (n=44) | TAU (n=43) | PP, FU (6mths) | Yes |
|  | Herring 2011, 2011a(2, 3) | USA | Community | RCT 3 groups | Women with a DSM-IV diagnosis of GAD (n=30) (M age=23.5) | No effect [RE-weight training 0.52; AE-cycling 0.04a] | RE-weight training (n=10); AE-cycling (n=10) | WL (n=10) | PP | NS |
|  | Hughes 2013(11) | USA | Community | RCT 2 groups | Adolescents with a DSM-IV diagnosis of MDD (n=30) (M age=17yrs) | No difference at post intervention (BG) [nr]. AE and stretching decreased depressive symptoms (PP) [0.36b], AE decreased symptoms more rapidly [0.21b] | AE (n=16); Stretching* (n=14) | None | PP, FU (6, 12mths) | No |
|  | Noorbakhsh, 2013(12) | Iran | University | RCT 3 groups | Female university students with mild-to-moderate depressive symptoms BDI (n=75) (M age=18.8yrs) | AE decreased depression (BG - PE) [nr] No difference between swimming and PE; swimming and AE. | AE (n=25); Swimming (n=25) | PE (n=25) | PP | NS |
|  | Olson, 2017(13) | USA | Comunity | RCT 2 groups | Young people with confirmed MDD diagnosis (n=50) (M age=21.1yrs) | AE decreased depression (BG) [0.14b] | AE (n=25) | Stretching (n=25) | PP | No |
|  | Parker 2016(4) | Australia | Community | 2x2 Factorial RCT 4 groups | Young people with mild-moderate anxiety and/or depression (n=176) (M age=17.6yrs) | PA decreased depression (BG) [BDI-II =0.41, MADRS= 0.48] | PA + PST (n=44); PA + SC (n=45) | PsyEd + PST (n=43); PsyEd + SC (n=44) | PP | Yes |
|  | Roshan 2011(14) | Iran | Secondary school | RCT 2 groups | Female adolescents scoring ≥18 on the HAM-D (n=24) (M age=16.9yrs) | AE-Pool walking decreased depression (BG) [nr] | AE-pool walking (n=12) | No intervention (n=12) | PP | NS |
|  | Sadeghi, 2016(15) | Iran | University | RCT 3 groups | University students with a depression diagnosis and BDI-II score between 13-28 (n=46) (M age=21yrs) | AE and CBT reduced depression (BG) [nr] | AE (n=16) | Group discussion (n=14);CBT (n=16) | PP | NS |
|  | Woolery, 2004(5) | USA | Community | RCT 2 groups | Volunteers with mild levels of depression BDI score between 10-15 (n=28) M age=21.5) | Yoga reduced depression (BG) [nr] | Yoga (n=13) | WC (n=15) | PP | NS |
|  | Wunram, 2018(16) | Germany | Psychiatric facility | Partly RCT 3 groups | Adolescent inpatients or day-clinic patients diagnosed with non-psychotic major depressive disorder and a DIKJ score >17 raw points (n=64) (M age=15.9 years) | AE – cycling ergometer reduced depression (BG AE – cycling ergometer – TAU control [endpoint =0.45, 26 weeks = 0.85]) No difference between AE – cycling ergometer and vibration plate at any time point. | AE – cycling ergometer + TAU (n=20) | Vibartion plate + TAU (n=21) | PP, FU (14wks & 26 weeks) | No* |
|  | Yavari, 2008(17) | Iran | University | RCT 2 groups | Male university students with BDI score >19 (n=74) (Age range=19-22) | AE – swimming reduced depression (BG) [nr] | AE – swimming (n=37) | No intervention (n=37) | PP | NS |
| Dysfunctional Attitudes | Sadeghi, 2016(15) | Iran | University | RCT 3 groups | University students with a depression diagnosis and BDI-II score between 13-28 (n=46) (M age=21yrs) | CBT reduced dysfunctional attitudes (BG) [nr] | AE (n=16) | Group discussion (n=14);CBT (n=16) | PP | NS |
| Eating Disorder Symptoms | Carei 2010(1) | USA | Community | RCT 2 groups | Adolescents diagnosed with eating disorders (n=53) (M age=16.5yrs) | Yoga decreased eating disorder symptoms (BG at 9-12 wks); Yoga decreased food preoccupation (PP) [0.16b] | Yoga (n=26) | WL (n=27) | PP, FU (4wks) | No* |
|  | Sundgot-Borgen 2002(18) | Norway | Community | RCT 4 groups | Adolescents with a DSM-IV diagnosis of BN (n=64) (M age=22.5yrs) | AE, CBT and Nutritional advice decreased bulimia, CBT and Nutritional advice decreased drive for thinness, AE and CBT decreased body dissatisfaction (PP). CBT decreased body dissatisfaction at PI and FU and bulimia and vomiting at FU (BG). AE reduced laxative use at PI and FU and drive for thinness, bulimic symptoms at FU (BG). AE and CBT reduced binges (PP). AE reduced binges compared to CBT at FU (BG). CBT reduced vomiting (PP) and at FU (BG). AE reduced laxative use (BG) [nr] | AE (n=15) | WL (n=16)CBT (n=16); Nutritional advice (n=17); | PP, FU (6, 18mths) | NS |
| Functioning | Curtis 2016(19) | Australia | Community | non-randomised CT 2 groups* | People diagnosed with first-episode psychosis (n=28) (M age=20.7yrs) | CE + health coaching + dietetic support + TAU improved health & social functioning (PP); CE + health coaching + dietetic support + TAU improved social occupational & psychological functioning (PP) [nr] | CE + health coaching + dietetic support + TAU (n=16) | TAU (n=12) | PP | Yes |
|  | Hughes 2013(11) | USA | Community | RCT 2 groups | Adolescents with a DSM-IV diagnosis of MDD (n=30) (M age=17yrs) | No difference at post intervention (BG). AE and stretching improved psychosocial functioning. [0.88b]. AE resulted in greater improvements in Family Global Assessment of Function (PP) [0.61b]. | AE (n=16); Stretching* (n=14) | None | PP, FU (6, 12mths) | No |
|  | Loh 2015(20) | Malaysia | Psychiatric facility | RCT 2 groups | Inpatients with a DSM-IV diagnosis of schizophrenia (n=104) (M age=21.6) | Walking improved personal and social performance (PP) no BG analysis presented]) [nr] | Walking (n=52) | TAU (n=52) | PP | Yes |
|  | Parker 2016(4) | Australia | Community | 2x2 Factorial RCT 4 groups | Young people with mild-moderate anxiety and/or depression (n=176) (M age=17.6yrs) | No effect [nr] | PA + PST (n=44); PA + SC (n=45) | PsyEd + PST (n=43); PsyEd + SC (n=44) | PP | Yes |
|  | Ventura 2013(7) | USA | Community | CT 2 groups (randomisation NS) | Individuals with first-episode schizophrenia (n=15) (M age=21yrs) | CTr+E improved school or work functioning [0.73], in independent living skills [1.26] and in family relationships [0.93] (BG) | CTr+E (n=ns) | TAU (n=ns) | PP | NS |
| Irritability | Herring 2011, 2011a(2, 3) | USA | Community | RCT 3 groups | Women with a DSM-IV diagnosis of GAD (n=30) (M age=23.5) | RE-weight training reduced irritability (BG) [Frequency - RE-weight training 1.18; AE-cycling 0.88; Intensity - RE-weight training 1.23; AE-cycling 0.74]a | RE-weight training (n=10); AE-cycling (n=10) | WL (n=10) | PP | NS |
| Mood States | Hughes 2013(11) | USA | Community | RCT 2 groups | Adolescents with a DSM-IV diagnosis of MDD (n=30) (M age=17yrs) | No difference at post intervention (BG). AE and stretching improved mood states (anger [0.26b], fatigue [0.47b] and tension [0.71b] (PP) | AE (n=16); Stretching* (n=14) | None | PP, FU (6, 12mths) | No |
|  | Brown 1992(9) | USA | Psychiatric facility | RCT 2 groups | Adolescents with dysthymia & conduct disorder (n=27) (M age=15.6yrs) | AE-Running improved mood states in girls (anxiety, hostility, confused thinking, and fatigue) (BG). AE-Running improved vigour (BG) [nr] | AE-running (n=17); Standard PE (n=10) | None | PP, FU (4wks) | No |
|  | Herring 2011, 2011a(2, 3) | USA | Community | RCT 3 groups | Women with a DSM-IV diagnosis of GAD (n=30) (M age=23.5) | RE-weight training reduced anxiety-tension (BG, wk 6) [RE-weight training 1.05; AE-cycling 0.73]a | RE-weight training (n=10); AE-cycling (n=10) | WL (n=10) | PP | NS |
| Distress | Jeong 2005(21) | Korea | Middle school | RCT 2 groups | Female adolescents with mild depression (n=40) (M age=16.0yrs) | Dance improved psychological symptoms & distress (BG) [nr] | Dance (n=20) | No intervention (n=20) | PP | NS |
| Negative Thoughts | Sadeghi, 2016(15) | Iran | University | RCT 3 groups | University students with a depression diagnosis and BDI-II score between 13-28 (n=46) (M age=21yrs) | CBT reduced belief in negative thoughts (BG)[nr] | AE (n=16) | Group discussion (n=14)  CBT (n=16); | PP | NS |
| Psychosis symptoms | Loh 2015(20) | Malaysia | Psychiatric facility | RCT 2 groups | Inpatients with a DSM-IV diagnosis of schizophrenia (n=104) (M age=21.6) | Walking improved psychosis symptoms (positive, negative symptoms and general psychopathology) (PP [no BG analysis presented]) [nr] | Walking (n=52) | TAU (n=52) | PP | Yes |
| Quality of Life | Carter 2015(10) | UK | Community | Pragmatic RCT 2 groups | Adolescents scoring >14 on the CDI-2 (n=87) (M age=15.4yrs) | No effect [nr] | TAU + CE-circuit training (n=44) | TAU (n=43) | PP, FU (6mths) | Yes |
|  | DelValle 2010(22) | Spain | Community | RCT 2 groups | Outpatients diagnosed with restrictive anorexia nervosa (n=22) (M age=14.5yrs) | No effect [nr] | RE-weight training (n=11) | No intervention (n=11) | PP | NS |
|  | Loh 2015(20) | Malaysia | Psychiatric facility | RCT 2 groups | Inpatients with a DSM-IV diagnosis of schizophrenia (n=104) (M age=21.6) | Walking improved QOL (physical functioning, physical role limitations, social functioning) (PP [no BG analysis presented]) [nr] | Walking (n=52) | TAU (n=52) | PP | Yes |
| Remission (Anxiety) | Herring 2011, 2011a(2, 3) | USA | Community | RCT 3 groups | Women with a DSM-IV diagnosis of GAD (n=30) (M age=23.5) | RE increased remission rate (BG) [nr] | RE-weight training (n=10); AE-cycling (n=10) | WL (n=10) | PP | Yes |
|  | Yang 2015(6) | China | Community | RCT 2 groups | University students diagnosed with anxiety disorder (n=38) (M age=21.8yrs) | CRT+ Counselling reduces anxiety recurrence compared to counselling (BG) [nr] | PA-CRT+Counselling (n=19) | Counselling (n=19) | PP, FU (3mths) | Yes |
| Remission (Depression) | Hughes 2013(11) | USA | Community | RCT 2 groups | Adolescents with a DSM-IV diagnosis of MDD (n=30) (M age=17yrs) | 100% at FU for AE; 70% at FU for stretching | AE (n=16); Stretching* (n=14) | None | PP, FU (6, 12mths) | No |
|  | Wunram | Germany | Psychiatric facility | Partly RCT 3 groups | Adolescent inpatients or day-clinic patients diagnosed with non-psychotic major depressive disorder and a DIKJ score >17 raw points (n=64) (M age=15.9 years) | No between group differences in remission rates at any time point | AE – cycling ergometer + TAU (n=20) | Vibration plate + TAU (n=21) | PP, FU (14wks & 26 weeks) | No* |
| Self-Efficacy | Brown 1992(9) | USA | Psychiatric facility | RCT 2 groups | Adolescents with dysthymia & conduct disorder (n=27) (M age=15.6yrs) | AE-Running improved self-efficacy (BG) [nr] | AE-running (n=17); Standard PE (n=10) | None | PP, FU (4wks) | No |
| Self-Esteem | Curtis 2016(19) | Australia | Community | non-randomised CT 2 groups* | People diagnosed with first-episode psychosis (n=28) (M age=20.7yrs) | No effect [nr] | CE + health coaching + dietetic support + TAU (n=16) | TAU (n=12) | PP | Yes |
| Sleep Quality | Curtis 2016(19) | Australia | Community | non-randomised CT 2 groups* | People diagnosed with first-episode psychosis (n=28) (M age=20.7yrs) | CE + health coaching + dietetic support + TAU improved sleep quality (PP) [nr] | CE + health coaching + dietetic support + TAU (n=16) | TAU (n=12) | PP | Yes |
| Social Adjustment | Hughes 2013(11) | USA | Community | RCT 2 groups | Adolescents with a DSM-IV diagnosis of MDD (n=30) (M age=17yrs) | No difference at post intervention (BG). AE and stretching improved social adjustment (except dating) (PP) [school =0.16b; friends = 0.19b; family =0.21b, anxiety = 0.43b] | AE (n=16); Stretching* (n=14) | None | PP, FU (6, 12mths) | No |
| Substance Use | Parker 2016(4) | Australia | Community | 2x2 Factorial RCT 4 groups | Young people with mild-moderate anxiety and/or depression (n=176) (M age=17.6yrs) | No effect [nr] | PA + PST (n=44); PA + SC (n=45) | PsyEd + PST (n=43); PsyEd + SC (n=44) | PP | Yes |
| Worry | Herring 2011, 2011a(2, 3) | USA | Community | RCT 3 groups | Women with a DSM-IV diagnosis of GAD (n=30) (M age=23.5) | Combined RE-weight training and AE-cycling conditions decreased worry (BG). No BG difference for intervention groups alone [RE-weight training 0.45, AE-cycling 0.45] a | RE-weight training (n=10); AE-cycling (n=10) | WL (n=10) | PP | Yes |

^a^ = Hedges’ d; AE=Aerobic Exercise; ^b^ = partial η^2^ BDI-II=Beck Depression Inventory-II; BN=Bulimia Nervosa; BG=Between Group Effects; CBT=Cognitive Behavioural Therapy; CBD: Cannot be Determined; CDI-(2)=Children’s Depression Inventory-(2); CE=combined aerobic + resistance; CRT=Collective Rehabilitation Training; CT=Controlled trial; CTr=Cognitive Training; DSM-IV=Diagnostic and Statistical Manual of Mental Disorders 4^th^ Edition; E=Exercise; FU=Follow Up; GAD= Generalised Anxiety Disorder; HAM-D= Hamilton Rating Scale of Depression; ITT=Intention to Treat Analysis; Min=Minutes; Mod=Moderate; n=sample size; NA=Not Applicable; NC=Nutritional Counselling; NS= Not stated; M=Mean; MDD=Major Depression Disorder; Mo=Months; nr=not reported; PA=Physical Activity; PE=Physical Education; PP=Pre-Post Intervention; PST=Problem Solving Therapy; PsyEd=Psychological Education; RCT= Randomised Control Trail; RE=Resistance Exercise; SC=Supportive Counselling; TAU=Treatment as Usual; VIG=Vigorous; Wks=Weeks; WL=Wait list; Yrs=Years; Stretching* We generally have classified stretching as a non-PA/E comparison intervention, but in this particular study the HR achieved by the stretching group was consistent with moderate intensity exercise. Therefore, in this study only, we classified stretching as a moderate intensity; No* states ITT but all randomised not included in analysis; Non-randomised CT 2 groups* unclear if two parallel groups, quasi-experimental or a controlled trial

Table 2: TIDiER table describing characteristics of the interventions

| **Ref** | **PA/E group** | **Exercise intensity/type** | **Non PA/E group** | **Personnel delivering treatment** | **Individual/group** | **Duration frequency** |
| --- | --- | --- | --- | --- | --- | --- |
| Balchin 2016(8) | AE-high: (cycling) Exercised at 70–75% of HR reserve; AE-mod: (cycling) Exercised at 45–50% of HR;  AE-low: kept their HRs below 120 bpm by walking and/or doing very light cycling | AE-high: VIGOROUS, AEROBIC;  AE-mod: MODERATE, AEROBIC;   AE-low: LIGHT, AEROBIC | NA | Not stated | Not stated | 60m 3xwk/6wks |
| Brown 1992(9) | AE: running/aerobic exercise program and continued in regularly scheduled physical activity classes;  Standard PE: regularly scheduled physical activity classes | AE: CBD [likely MODERATE to VIGOROUS], AEROBIC; Standard PE: CBD [likely MODERATE to VIGOROUS] | NA | Not stated | Not stated | (duration ns) AE: 3xwk/9wk |
| Carei 2010(1) | Yoga: Yoga sessions followed a yoga treatment manual. All participants received standard medical care regardless of group assignment | Yoga: CBD [likely LIGHT to MODERATE] | WL: All participants received standard medical care regardless of group assignment | Certified yoga instructor | Individual | 60m 2xwk/8wk |
| Carter 2015(10) | TAU + CE: (circuit training): Interval pattern with 8 separate exercise-stations. The stations consisted of strengthening and aerobic exercises: abdominal and back exercises from the supine and prone positions respectively; 2 medicine ball arm-based exercises from supine position; bouncing, static and dynamic balance exercises on a trampoline; body-Weight squat exercise against the wall and stationary cycling. Following 5mins of stretching, participants were encouraged to exercise for 1min then break for 1min, this was then repeated twice more. Subsequently, participants exercised for 2min followed by a break of 1min; this was then repeated 9 times. Subsequently, a 5min stretching exercise closed the intervention. | CE: LIGHT-MOD [participant preference], AEROBIC/RESISTANCE | TAU | Qualified exercise therapist | Group | 60m 2xwk/6wks |
| Curtis 2016(19) | Enhanced PE: The first step included minimum activity with no weight transfer: stretching, and nonstrenuous arm, leg and trunk movement. The second step included weight transfer activities and incorporated dynamic large muscle movements such as fast walk | CE: MODERATE to VIGOROUS, AEROBIC/RESISTANCE | TAU | Specialist clinical staff (nurse, dietician and exercise physiologist) and youth peer wellness coaches | Individual | (frequency, duration ns) 12wks |
| DelValle 2010(22) | RE: (weight training): Each session started and ended with a low-intensity warm-up and cool-down period (10–15 mins each), each consisting of stretching exercises involving all major muscle groups. The core portion of the session included 11 strength exercises engaging the major muscle groups, that is, bench press, shoulder press, leg extension, leg press, leg curl, abdominal crunch, low back extension, arm curl, elbow extension, seated row, and lateral pull-down. The participants performed 1 set of 10–15 reps until volitional fatigue per exercise, with resting periods of 1–2mins... Participants also performed isometric contractions of large muscle groups (six sets of three repetitions each, 20–30-s duration per repetition) with their own body weight (for lower body exercises) or barbells (1–3 kg) for upper body | RE: CBD, RESISTANCE [LIKELY MOD-VIG] | No intervention | Instructor | Group | 60-70m 2xwk/12wks |
| Herring 2011, 2011a(2, 3) | RE: (weight training): 7sets of 10reps were performed of leg press, leg curl and leg extension exercises beginning at 50% of the predicted 1-RM during wk 1 and progressing by 5% of the predicted 1-RM weekly. Each exercise was preceded by a warm-up set of 10 eps beginning at 35% of the predicted 1-RM during week 1 and progressing by 5% of the predicted 1-RM weekly. Each eccentric and concentric action was performed for 2s so that each set required 40s; AE: (cycling): 2 weekly sessions of 16min of continuous leg cycling were performed | RE: VIGOROUS, RESISTANCE; AE: LIGHT, AEROBIC | WL | Exercise specialists | Not stated | RE: 46m 2xwk/6wks; AE: 16m 2xwk/6wks |
| Hughes 2013(11) | AE: Exercise program: "Supervised exercise sessions at the Cooper Institute for the participants began by using the treadmills or stationary cycles. The CI trainers also taught patients how to complete home-based exercise sessions (e.g., choice of Wii Sports and Fit, jazz exercise, jogging, weight training based on their preferred exercise) that were unsupervised workouts at the patient’s home or in the community Stretching: The series included such traditional “warm-up” stretches as: stretches of the gluts, inner thigh, calves and ankles, Achilles tendon, hamstring stretches, shoulder rolls forward and back, shoulder shrugs, isometrics for the neck hugging knees into the chest, moving forehead to right knee, then to left, then to both, and use of the pelvic tilt. An additional 10-15min consisted of moving on to right and left calf stretches, quad stretches, and then to a series for the arms, hands, fingers, wrist, biceps/triceps, shoulders and back. All of the exercises were designed to be done slowly, emphasizing proper alignment, and rest periods to minimize overall physical exertion while obtaining general flexibility. After 2 weeks of 3 sessions at CI they moved to once a week at CI and 2 home-based sessions | AE: VIGOROUS, AEROBIC; Stretching: MODERATE [aimed to be LIGHT] | NA | Cooper Institute trainers | Not stated | 30-40m 3xwk/12wks |
| Jeong 2005(21) | Dance: Sessions were designed around 4 major themes: (a) awareness of the body, the room, and the group; (b) movement expressions and symbolic quality of movement; (c) movement, feeling, images, and words; and (d) differentiation and integration of feelings. Each of these themes included various sub-themes: (a) setting limits and outer, inner, and personal space; (b) body language, the reflecting process, polarity, and inward and outward expression; (c) playing, drawing, and verbalization; and (d) the inner sense, quality of movement, and expression of feelings | Dance: CBD [likely LIGHT-MOD] | No intervention | Not stated | Not stated | 45m 3xwk/12wks |
| Loh 2015(20) | Walking: In the first month, participants partake in a 20-minute walking exercise per session with 5-minute warm-up and 5-minute warm down sessions. In the second month, the session increased to 30-minutes walking exercise per session with 5-minute warm-up and 5-minute warm down sessions. In the third month, the session increased to 40-minute walking exercise with 5-minute warm-up and 5-minute warm down sessions | Walking; CBD [likely MODERATE], AEROBIC | TAU | Supervised by ward staff/medical officers | Group | 20-40m 3xwk/12wks |
| Parker 2016(4) | PA: This intervention was based on behavioural activation principles. Participants were provided with psychoeducation on the relationship between exercise and mood/anxiety symptoms, government guidelines for physical activity a costs and benefits worksheet about engaging in physical activity, physical activity diaries and pedometers for motivational purposes.The type of physical activity was not prescribed; rather physical activities were tailored and chosen based on theindividual participant’s interests, prior activities that were enjoyable or offered a sense of achievement, current activity or perceived fitness levels, resources and social supports. The intervention included weekly goal setting, focusing on incremental changes and including incidental activities; PsychoEd (incl. PA): This intervention provided the same psychoeducation and resources as the behavioural activation intervention, as well as weekly resources focusing on sleep, substance use, and other lifestyle information. This was designed to match weekly session time spent on the intervention in the active group. There sources were discussed in terms of general utility of the content of each, but the therapists did not specifically engage with participants on how to act on the information provided. The importance of physical activity was addressed in the first session but was not included in ongoing intervention | PA: CBD [participant preference] | SC: The intervention was based on general counselling principles and was informed by the NICE guidelines for young people with mild to moderate depression The main goals of the intervention were to engage and build rapport so that the young person felt as though their concerns had been heard, that some 1 appreciated their experience and to work together on addressing current difficulties; PST: The intervention progressively worked through the 7 steps of PST, namely: (1) identifying the young person’s problem/s; (2) selecting 1 or 2 key problems; (3) identifying and operationalising goals; (4) brainstorming and generating solutions;(5 exploring the risks and benefits of solutions and choosing a solution; (6) creating a SMART(specific, measurable, achievable, relevant, time-limited) plan, and (7) re-viewing progress/evaluating the plan | Research psychologists | Individual | (duration variable) 1xwk/6wks |
| Roshan 2011(14) | AE: The pool walking exercise was carried out in a pool with 15 meters width. The water height in the pool was considered as much as 70 to 80% of the cases' height, and they walked with respect of their height in determined water height. The activity intensity was constantly about 60-70% of maximum heart rate. On the average, the cases walked every 30-meter distances with aforesaid intensity in 50 to 60 seconds. The relaxation time between every walking was 30 to 40 seconds. The break time between the sets was 5 to 6 mins in order to allow heart rate to return to primary situation | AE: MODERATE, AEROBIC | No intervention | Not stated | Not stated | (duration ns) 3xwk/6wks |
| Sadeghi, 2016(15) | AE: Typically, the movements started from the head, neck or legs and continued by running in place. This stage took about 10 minutes. Then, the movements were carried on with greater intensity. The average intensity of movements was 0.60 to 0.80 heart rate. This step was faster with gestures and movements of the hands and feet separately, one-way, two-way, and cross legs. At this stage, the heart rate was measured and recorded by Radial or Carotid pulse. Duration of this period was 30 to 35 minutes. The final stage was the cooling down stage with less intensity, lasting for almost 10-15 minutes | AE: CBD [likely MOD-VIG] | CBT: The cognitive therapy group received 12 sessions of cognitive behavior therapy, 2 sessions per week, in the first half of treatment, and 1 session per week for the second half based on the cognitive model of Michael Frey; Control: The members of the control group gathered in a classroom or the amphitheater of the Faculty of Health at KUMS. They tried to discuss the issues raised by themselves | Sport Instructor | Not stated | AE: 45-60m/Frequency ns/8wks; CBT: 45-60m/2xwk/8wks; Control: 45-60m/Frequency ns/8wks |
| Sundgot-Borgen 2002(18) | AE: The aerobic activity level was calibrated to keep participants at 50–70% of their maximal oxygen consumption (45 min of jogging, cross-country skiing, or swimming) followed by a 15-min cool down and stretching. Within the treatment program, subjects were advised to exercise at least 35 min 2 times·wk 1 without the instructor being present | AE: MODERATE to VIGOUROUS, AEROBIC | NC: Meal planning was introduced to establish and maintain a pattern of regular eating; CBT: The aim of CBT was (a) to enable patients to identify thoughts, feelings, or events before or during bulimic episodes and thereby to discover how bingeing and purging may soothe or regulate emotions; (b) to enable patients to identify and modify core beliefs that perpetuate bulimic behavior; (c) to introduce behavioral techniques to combat urges to binge or vomit, and to develop alternatives to bulimic eating patterns to cope with disturbing thoughts and emotions; and (d) to provide training in general problem-solving skills | AE: Fitness instructor; NC: Registered dietitian; CBT: Therapist | Group | AE: 60m 1xwk/16wks + 35m 2xwk/16wks w/ out instructor; NC: 120m 2xwk/2wks then 120m 1xwk/14wks; CBT: 120m 1xwk/16wks; |
| Ventura 2013(7) | CTr + E: computerized brain plasticity-based training focused on auditory discrimination and then switched to computerized social cognition training. These same patients exercised for 30 mins twice a week at the clinic and for 30 mins at home | E: CBD | Aftercare TAU: Involving a healthy lifestyle psychoeducational group | Not stated | Not stated | 30m 3xwk/10wks |
| Yang 2015(6) | PA-CRT + Counselling: encouraging patients to participate in collective outdoor games, for example, joining in the collective game consisting of about 20 people. The main game is the entertainment 1, which aims at the team cooperation. As for the weekly activities, the main 1 is the collective training project, additionally; 1-2 short distance travels, hiking and picnics can be arranged | PA: CBD [likely LIGHT to MODERATE] | Counselling: At the early stage of the psychological counseling, in order to be trusted by the patients, the psychological support therapy is adopted, for example, explaination, encourage, comfort and guarantee methods. It is required that the doctor shall communicate with the patient in the guidance way and understand the disease reasons and sources of the patient by the means of listening in the process of communication. In the middle-late stage of the psychological counseling, the main therapies are cognitive counseling, narrative therapy, psychological counseling and behavioral therapy | Not stated | Group | PA-CRT: ≥60m (depending on activity) 3-5wk/8wks; Counselling: 30m 2xwk/8wks |
| Noorbakhsh, 2013(12) | AE; Swimming | AE - CBD[ likely MOD-VIG], AEROBIC | Classes as usual – Physical Education | Not stated | Not stated | 60mins 3xwk/6wks |
| Olson, 2017(13) | AE: AE consisted of 45 min of continuous steady-state exercise performed on a treadmill or cycle ergometer at a prescribed moderate-intensity corresponding to 40–65% of HR reserve (HRR), which was determined from HR recorded during the initial baseline fitness test. Participants were instructed and encouraged to maintain this intensity during all exercise sessions. This dose of exercise is consistent with public health recommendations and has been shown to increase the likelihood of successful adherence. | AE – MOD-VIG, AEROBIC | Attention control - Stretching | Trained laboratory staff | Not stated | 30-45mins 3xwk/8wks |
| Woolery, 2004(5) | Yoga: Subjects were taught the Iyengar approach to yoga. Classes emphasized postures that, according to the Iyengar yoga perspective, are supposed to alleviate depression, particularly back bends, standing poses, and inversions. Classes ended with relaxation postures that open the chest. All subjects were taught the same asanas, the one exception being that menstruating women practiced alternatives to inversions | Yoga: CBD [likely LIGHT to MODERATE] | WL | Certified Iyengar yoga teacher | Group | 60 mins 2xwk/5wks |
| Wunram | EA - Cycling ergometer + TAU: The ergometer training took place on stationary cycles. A 30-min interval training calculated on the maximal performance in the previous spiroergometry results was applied. TAU followed participants therapy schedule at the inpatient units. Common therapy offers were psychotherapy in form of individual sessions with a psychotherapist or psychiatrist, group psychotherapy sessions, exercise therapy, art therapy and music therapy. | Cycling ergometer: VIGOROUS, AEROBIC | Vibration plate: The vibration plate stimulates a movement pattern similar to human gait. The training principle is based on the activation of proprioceptive spinal circuits, inducing a certain number of stretch reflex contractions per second depending of the frequency chosen. The training improves muscle power and function and coordination of the legs and the hip and also partly of the trunk, having only a small effect on the cardiovascular system. Besides that it improves bone formation and the metabolism in skeletal muscles and skin. | Study personnel | Group and individual (range 1-6 participants) | Cycling ergometer: 30mins Vibration plate: 6 exercises x 2-3mins 1-2xwk/6wks |
| Yavari, 2008(17) | AE - Swimming | AE swimming CBD [likely MOD-VIG], AEROBIC | No intervention | Not stated | Not stated | 1xwk/12-15wks |

AE=Aerobic Exercise; CBT=Cognitive Behavioural Therapy; CBD: Cannot be Determined; CE=combined aerobic + resistance; CRT=Collective Rehabilitation Training; CT=Cognitive Training; E=Exercise; HR=Heart Rate; M=Minutes; MOD=Moderate; NA=Not Applicable; NC=Nutritional Counselling; NS= Not stated; PA=Physical Activity; PE=Physical Education; PST=Problem Solving Therapy; PsyEd=Psychological Education; RE=Resistance Exercise; SC=Supportive Counselling; TAU=Treatment as Usual; VIG=Vigorous; Wk=Week; WL=Wait list.

Supplementary Table 3. Search strategy used in the “Evidence Finder.”

| **Step 1. Mental health or substance use problem** | **Step 2. Stage of illness** | **Step 3. Treatment / Intervention** | **Step 3a. Treatment / Intervention** | **4. Publication date** | **5. Keywords** | **Advanced Options** |
| --- | --- | --- | --- | --- | --- | --- |
| Anxiety disorders (any) | At risk (indicated or selected prevention) | Biological interventions (any) | Acupuncture/acupressure | 1980-2019 | None | **Systematic reviews** |
| Bipolar disorders | Disorder established (diagnosed disorder) | **Complementary & alternative interventions (CAM)** | Bright light therapy |  |  | **Randomised control trials** |
| Depressive disorders | First episode (psychosis only) | Psychological interventions (any) | Creative expression: music, dance, drama, art |  |  | **Controlled clinical trails** |
| Eating disorders (any) | Relapse prevention | Service delivery and improvement | Dietary advice/dietary changes |  |  |  |
| Psychosis disorders | Treatment resistant/treatment refractory |  | Homeopathic, plant based medicines |  |  |  |
| Substance use disorders (any) | Universal prevention |  | Massage |  |  |  |
| Suicide and self-harm (any) |  |  | Meditation |  |  |  |
|  |  |  | Mind-body exercises (e.g. yoga, tai chi, qigong) |  |  |  |
|  |  |  | Omega 3 fatty acids (e.g. fish oil, flax oil) |  |  |  |
|  |  |  | Other complementary & alternative interventions |  |  |  |
|  |  |  | **Physical activity/exercise** |  |  |  |
|  |  |  | Relaxation |  |  |  |
|  |  |  | Vitamins and supplements |  |  |  |

Bold writing indicates where a filter has been applied. No filters were applied to steps 1, 2 and 5.

Supplementary Table 4. Risk of bias assessment for included studies

| Study | Allocation concealment | Sequence Generation | Blinding of outcome assessors | Incomplete outcome data | Selective outcome reporting |
| --- | --- | --- | --- | --- | --- |
| Balchin 2016 | unclear | unclear | low | low | unclear |
| Brown 1992 | unclear | unclear | unclear | high | unclear |
| Carei 2010 | low | low | low | low | unclear |
| Carter 2015 | low | low | low | low | low |
| Curtis 2016 | NA | NA | unclear | high | unclear |
| delValle 2010 | low | unclear | unclear | unclear | high |
| Herring 2011 | unclear | unclear | unclear | unclear | low |
| Herring 2011 | low | low | low | low | unclear |
| Hughes 2013 | low | low | low | unclear | low |
| Jeong 2005 | low | unclear | unclear | low | unclear |
| Loh 2015 | low | low | unclear | low | unclear |
| Noorbakhsh 2013 | unclear | unclear | unclear | unclear | unclear |
| Olson 2017 | low | low | unclear | low | unclear |
| Parker 2016 | low | low | low | low | low |
| Roshan 2011 | unclear | unclear | unclear | unclear | low |
| Sadeghi 2016 | unclear | unclear | unclear | unclear | unclear |
| Sundgot-Borgen 2002 | unclear | unclear | low | low | unclear |
| Ventura 2013 | unclear | unclear | unclear | unclear | unclear |
| Woolery 2004 | unclear | unclear | unclear | unclear | unclear |
| Wunram 2018 | low | low | low | low | high |
| Yang 2015 | low | low | unclear | unclear | unclear |
| Yavari 2008 | unclear | unclear | unclear | unclear | unclear |

Sequence Generation: UC = method of randomisation not stated, NA=Not applicable as study not randomised; Allocation: UC = does not specify if allocation concealment maintained, NA=Not applicable as study not randomised; Blinding of outcome assessor: UC = not specified if outcome assessor blind or not or if blinding likely to affect outcome result; Incomplete outcome data: UC = drop out numbers and/or reasons in each group not stated; H = very high dropout rates or reasons for drop out seem to differ between groups, or excluded one participant due to non-adherence; Selective outcome reporting: UC = as no protocol paper; H = an outcome in protocol paper does not seem to be reported.

1. Carei TR, Fyfe-Johnson AL, Breuner CC, Brown MA. Randomized controlled clinical trial of yoga in the treatment of eating disorders. J Adolesc Health. 2010;46(4):346-51.

2. Herring MP, Jacob ML, Suveg C, Dishman RK, O'Connor PJ. Feasibility of exercise training for the short-term treatment of generalized anxiety disorder: A randomized controlled trial. Psychotherapy & Psychosomatics. 2011;81(1):21-8.

3. Herring MP, Jacob ML, Suveg C, O’Connor PJ. Effects of short-term exercise training on signs and symptoms of generalized anxiety disorder. Mental Health and Physical Activity. 2011;4(2):71-7.

4. Parker AG, Hetrick SE, Jorm AF, Mackinnon AJ, McGorry PD, Yung AR, et al. The effectiveness of simple psychological and physical activity interventions for high prevalence mental health problems in young people: A factorial randomised controlled trial. Journal of affective disorders. 2016;196:200-9.

5. Woolery A, Myers H, Sternlieb B, Zeltzer L. A yoga intervention for young adults with elevated symptoms of depression. Alternative therapies in health and medicine. 2004;10(2):60-3.

6. Yang WL, Zhai F, Gao YM, Zhang QH. Collective rehabilitation training conductive to improve psychotherapy of college students with anxiety disorder. Int J Clin Exp Med. 2015;8(6):9949-54.

7. Ventura J, Gretchen-Doorly, D., Subotnik, K. L., Vinogradov. S., Nahum, M., Nuechterlein, K. H. Combining cognitive training and exercise to improve cognition and functional outcomes in the early course of schizophrenia: A pilot study. Abstracts for the 14th International Congress on Schizophrenia Research (ICOSR): Schizophrenia Bulletin; 2013. p. S1–S358.

8. Balchin R, Linde J, Blackhurst D, Rauch HL, Schonbachler G. Sweating away depression? The impact of intensive exercise on depression. Journal of affective disorders. 2016;200:218-21.

9. Brown SW, Welsh MC, Labbe EE, Vitulli WF, Kulkarni P. Aerobic exercise in the psychological treatment of adolescents. Percept Mot Skills. 1992;74(2):555-60.

10. Carter T, Guo B, Turner D, Morres I, Khalil E, Brighton E, et al. Preferred intensity exercise for adolescents receiving treatment for depression: a pragmatic randomised controlled trial. BMC psychiatry. 2015;15:247.

11. Hughes CW, Barnes S, Barnes C, DeFina LE, Nakonezny P, Emslie GJ. Depressed Adolescents Treated with Exercise (DATE): A pilot randomized controlled trial to test feasibility and establish preliminary effect sizes. Mental Health and Physical Activity. 2013;6(2):119-31.

12. Noorbakhsh M, Alijani E. The effects of physical activity on the level of depression in university female students. Annals of Biological Research. 2013;4(8).

13. Olson RL, Brush CJ, Ehmann PJ, Alderman BL. A randomized trial of aerobic exercise on cognitive control in major depression. Clinical neurophysiology : official journal of the International Federation of Clinical Neurophysiology. 2017;128(6):903-13.

14. Roshan VD, Pourasghar M, Mohammadian Z. The efficacy of intermittent walking in water on the rate of MHPG sulfate and the severity of depression. Iranian Journal of Psychiatry and Behavioural Science. 2011;5(2):26-31.

15. Sadeghi K, Ahmadi SM, Ahmadi SM, Rezaei M, Miri J, Abdi A, et al. A Comparative Study of the Efficacy of Cognitive Group Therapy and Aerobic Exercise in the Treatment of Depression among the Students. Glob J Health Sci. 2016;8(10):54171.

16. Wunram HL, Hamacher S, Hellmich M, Volk M, Janicke F, Reinhard F, et al. Whole body vibration added to treatment as usual is effective in adolescents with depression: a partly randomized, three-armed clinical trial in inpatients. European Child & Adolescent Psychiatry. 2018;27(5):645-62.

17. Yavari A. The effect of swimming in reduction of depression in university male students. Research Journal of Biological Sciences. 2008;3(6):543-5.

18. Sundgot-Borgen J, Rosenvinge JH, Bahr R, Schneider LS. The effect of exercise, cognitive therapy, and nutritional counseling in treating bulimia nervosa. Medicine and Science in Sports and Exercise. 2002;34(2):190-5.

19. Curtis J, Watkins A, Rosenbaum S, Teasdale S, Kalucy M, Samaras K, et al. Evaluating an individualized lifestyle and life skills intervention to prevent antipsychotic-induced weight gain in first-episode psychosis. Early Interv Psychiatry. 2016;10(3):267-76.

20. Loh SY, Abdullah A, Abu Bakar AK, Thambu M, Nik Jaafar NR. Structured Walking and Chronic Institutionalized Schizophrenia Inmates: A pilot RCT Study on Quality of Life. Glob J Health Sci. 2015;8(1):238-48.

21. Jeong YJ, Hong SC, Lee MS, Park MC, Kim YK, Suh CM. Dance movement therapy improves emotional responses and modulates neurohormones in adolescents with mild depression. Int J Neurosci. 2005;115(12):1711-20.

22. del Valle MF, Perez M, Santana-Sosa E, Fiuza-Luces C, Bustamante-Ara N, Gallardo C, et al. Does resistance training improve the functional capacity and well being of very young anorexic patients? A randomized controlled trial. J Adolesc Health. 2010;46(4):352-8.
